# Supplementary material for: Krüppel-like Transcription Factor 7 Is a Causal Gene in Autism Development
Source: Int J Mol Sci. 2022 Mar 21;23(6):3376. doi: 10.3390/ijms23063376 (PMC8949233; doi:10.3390/ijms23063376)
Supplement: Supplementary file 1 [file ijms-23-03376-s001.zip › ijms-1607857-supplementary figure.pdf]

Supplementary Materials:

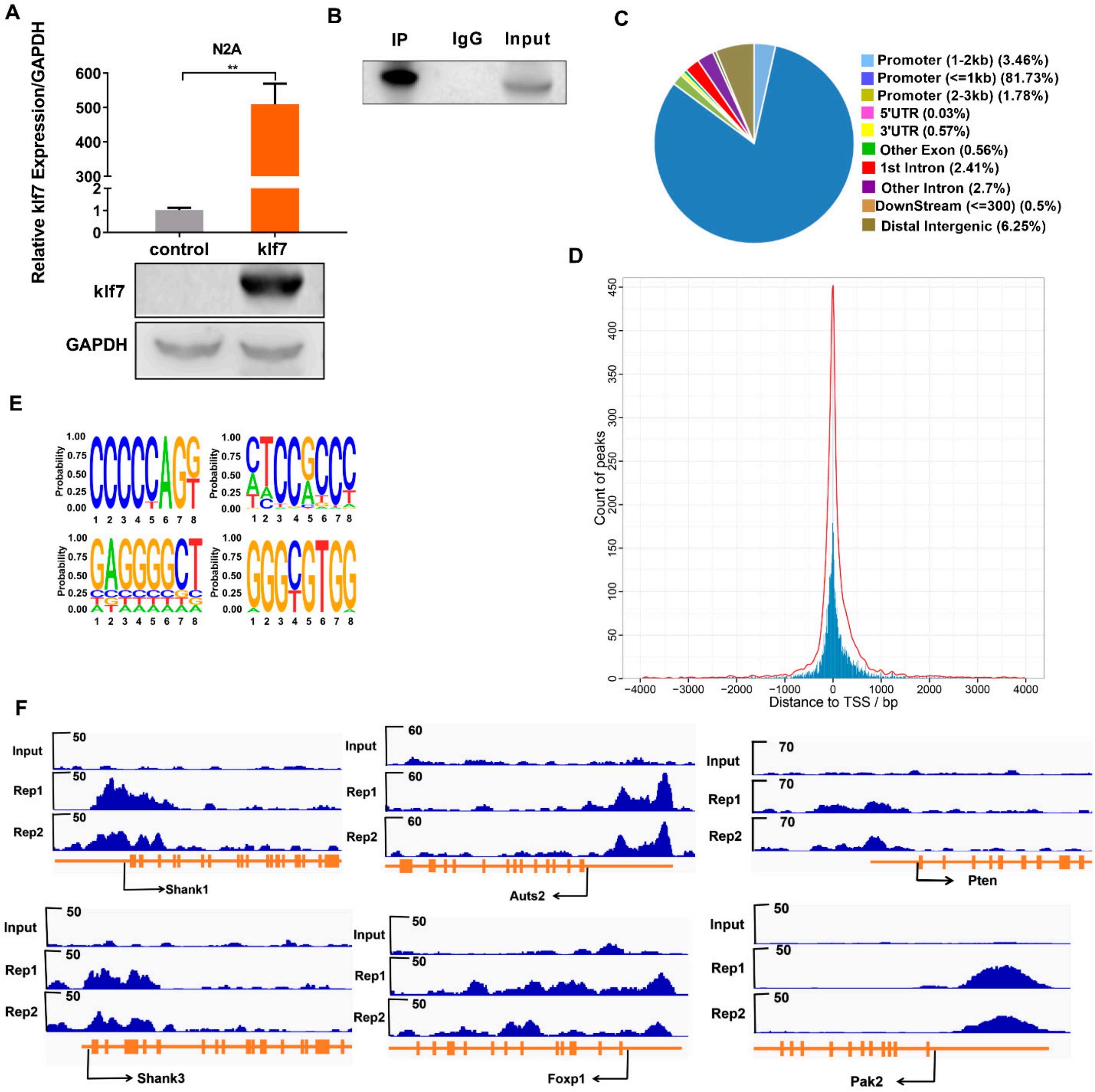

Supplemental Figure S1: Genome binding of klf7, as determined by ChIP-seq (related to Figure 1). (A) The mRNA and protein levels of klf7 in N2A cells transfected with empty vector or HA-klf7 expression vector. The results of qRT-PCR with primers specific for klf7 and a representative western blot for HA revealing increased klf7 expression levels in N2A cells. (B) Anti-HA antibody validation. Representative immunoprecipitation of N2A extracts with an anti-HA antibody followed by western blotting. A parallel test was performed with rabbit IgG as a control. (C) The distribution of peaks bound by klf7 in functional gene regions. (D) Peaks bound by klf7 existed at approximately ± 1kb

from the TSSs. (E) Motif enrichment analysis for klf7-bound peaks. (F) Binding peaks for several high-confidence ASD risk gene were identified in two biological replicates by Chip-seq. ChIP-seq, chromatin immunoprecipitation sequence; mRNA, messenger RNA; klf7, krüppel-like transcription factor 7; qRT-PCR, quantitative realtime polymerase chain reaction; TSSs, transcription start sites; ASD, autism spectrum disorder. ** $p < 0.01$.

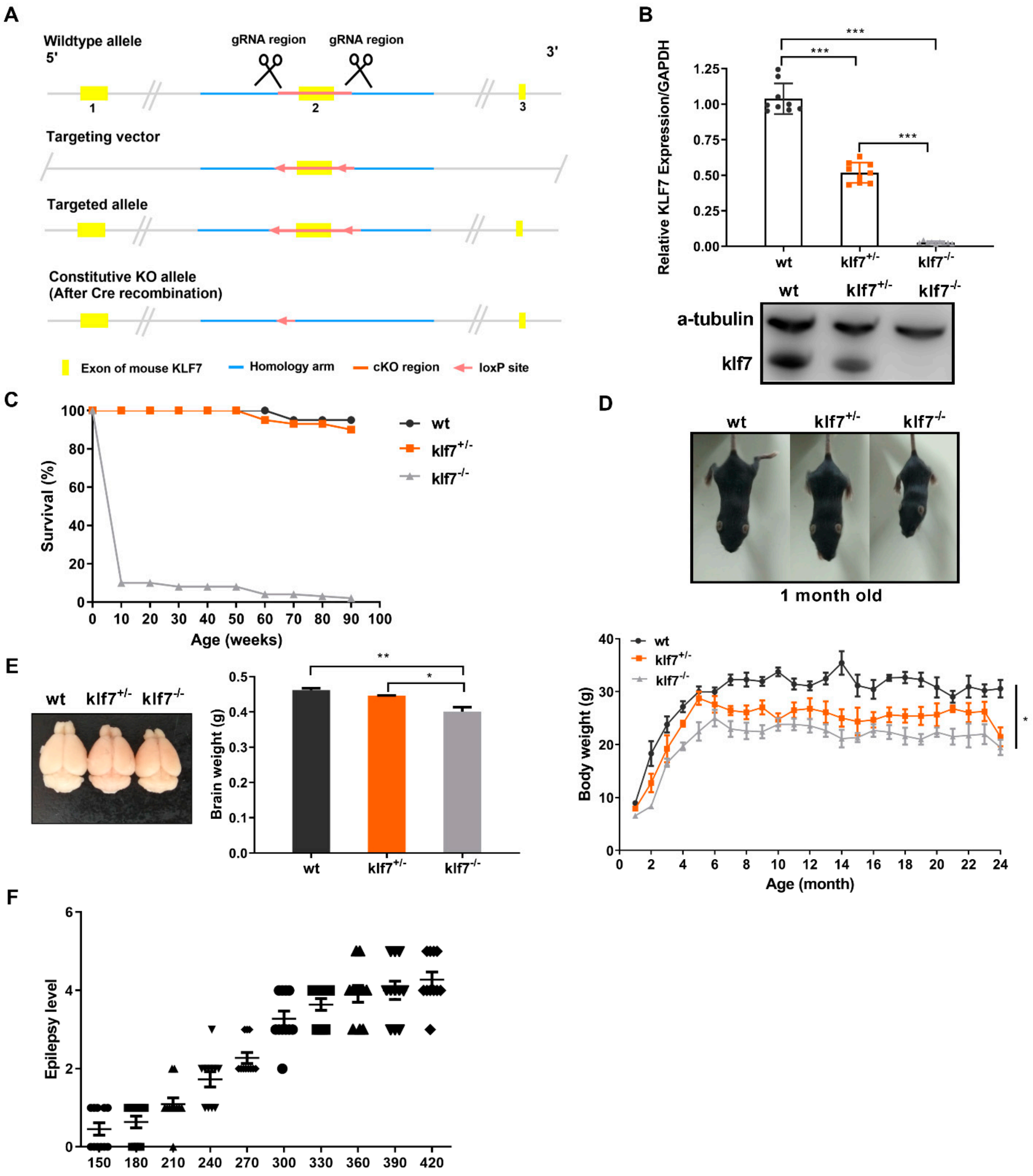

Supplemental_Fig_S2: Effects of klf7 deficiency in adult mice (related to Figure 2)

(A) Schematic diagram of the klf7 knockout strategy. The WT locus was inserted before and after the loxp site. (B) The mRNA expression level and protein level of klf7 were decreased in the whole brain from 1-month-old WT, klf7+/-, and klf7-/- mice. Klf7 expression was reduced by 47% in klf7+/- mice and by 97% in klf7-/- mice; n = 9 for each group of qRT−PCR and n=4 for each group of western blot.

(C) Postnatal survival rates of WT (n = 30), klf7+/- (n = 32), and klf7-/- (n = 20) mice. WT mice and klf7+/- mice survived for up to at least 90 weeks, and klf7-/- mice had a survival rate of less than 10% by 10 weeks of age. (D) Male WT mice, littermate klf7+/-, and littermate klf7-/- mice at 1 month of age. Histogram (right) of the body weights of WT (n = 29), littermate klf7+/- (n = 31), and littermate klf7-/- (n = 12) mice. (E) Brain weights of WT (n = 24), littermate klf7+/- (n = 20), and littermate klf7-/- (n = 15) mice. (F) Epilepsy-related behaviors of klf7+/- mice. The mice first developed seizures at approximately 4 months of age, and seizures peaked at approximately 1 year of age; n = 30 for assessment of epilepsy-related behaviors. The data are presented as the mean ± SEM. $P^{*}<0.05$, $P^{**}<0.01$ and $P^{***}<0.001$ by one-way ANOVA. klf7, krüppel-like transcription factor 7; WT, wild type;

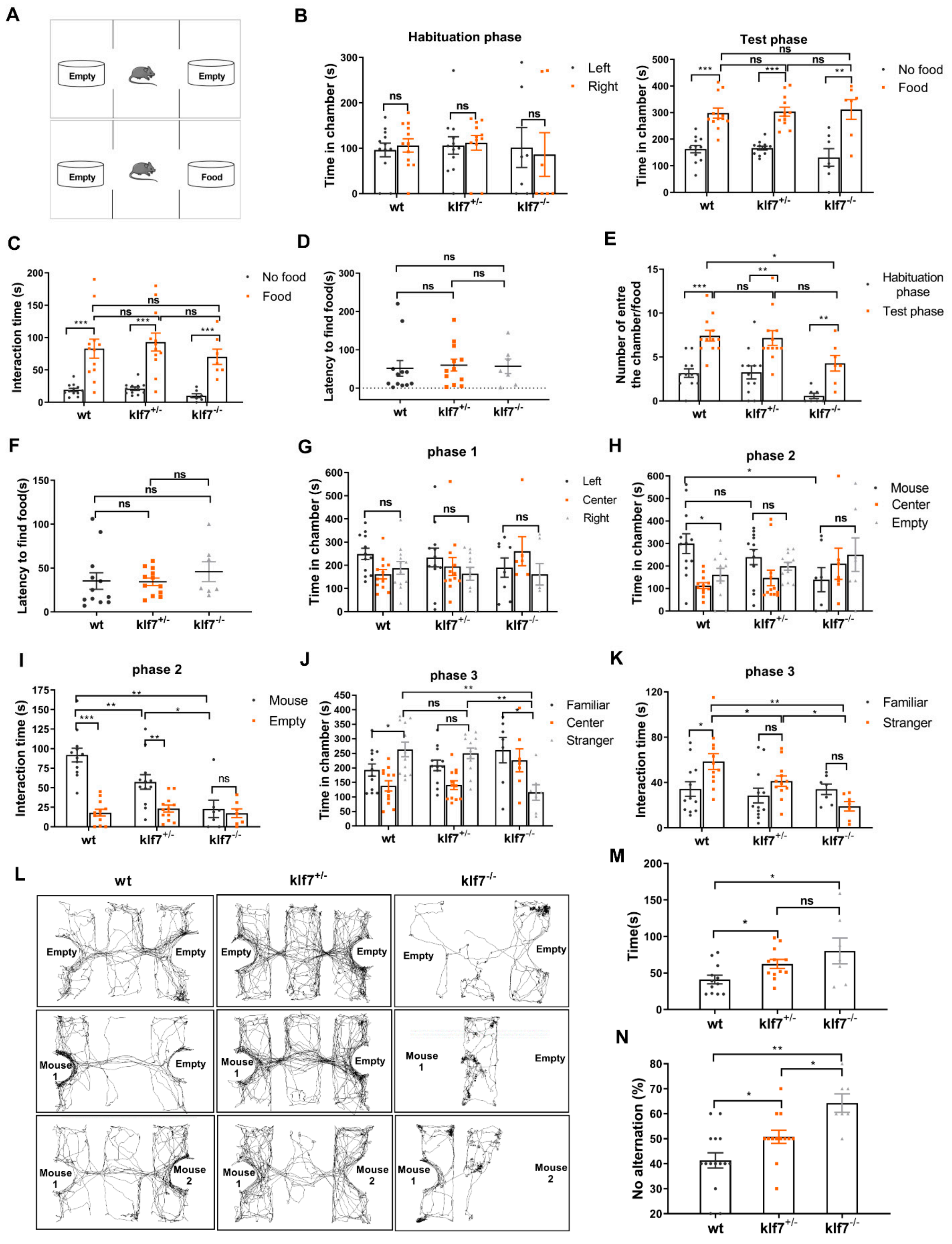

Supplemental_Fig_S3: klf7 deletion amplifies the core symptoms of ASD (related to Figure 2).

(A-E) Olfactory was assessed by a modified three-chamber experiment. (A) Diagram of the modified three-chamber olfactory test. (B) Time spent in the different chambers in the experiment. During the habituation phase, there was no significant difference in the time spent in the different chambers. During the testing phase, mice of all genotypes mice preferred to stay in the food-containing chamber. (C) Interaction time. Mice of all genotypes preferred to interact with food-containing dishes. (D) Latency to find food for the first time. Mice of all genotypes found food in a short amount of time, with no difference between the groups. (E) Number of entries into the food-containing chamber. Mice of all genotypes mice entered the food-containing chamber more frequently in the test phage than in the habituation phase. (F) Food-burying test. Mice of all genotypes found food quickly. (G-L) Three chamber experiment. (G) Mice of all genotypes showed no preference for the two empty chambers. (H) While WT mice spent more time in the chamber containing stranger mouse 1, klf7+/- and klf7-/- mice had no preference for either chamber, especially klf7-/- mice. (I) The time spent interacting with stranger mouse 1 by klf7+/- and klf7-/- mice was decreased compared with that by WT mice. (J) While WT mice spend more time in the novel mouse-containing chamber than in the familiar mouse-containing chamber, klf7+/- and klf7-/- mice showed no preference for either chamber. (K) klf7+/- and klf7-/- mice interacted with the novel mouse for a shorter amount of time than WT mice. (L) Tracks of mice of all genotype mice in the social interaction experiment. (M-N) klf7+/- and klf7-/- mice exhibited stereotypical behaviors. (M) klf7+/- and klf7-/- mice spent more time in self-grooming than WT mice. (N) In the Y maze spontaneous selection experiment, klf7+/- and klf7-/- mice preferred the original target arm, while WT mice preferred to explore the novel arm. The is indicated by the ratio of entries into the original target arm to entries into the novel arm in 10 tests. The data are presented as the mean ± SEM. n = 12 for WT and klf7+/- mice, n=7 for klf7-/- mice. $P^{*}<0.05$, $P^{**}<0.01$, $P^{***}<0.001$ by one-way ANOVA and two-way ANOVA. klf7, krüppel-like transcription factor 7; ASD, autism spectrum disorder; WT, wild type.

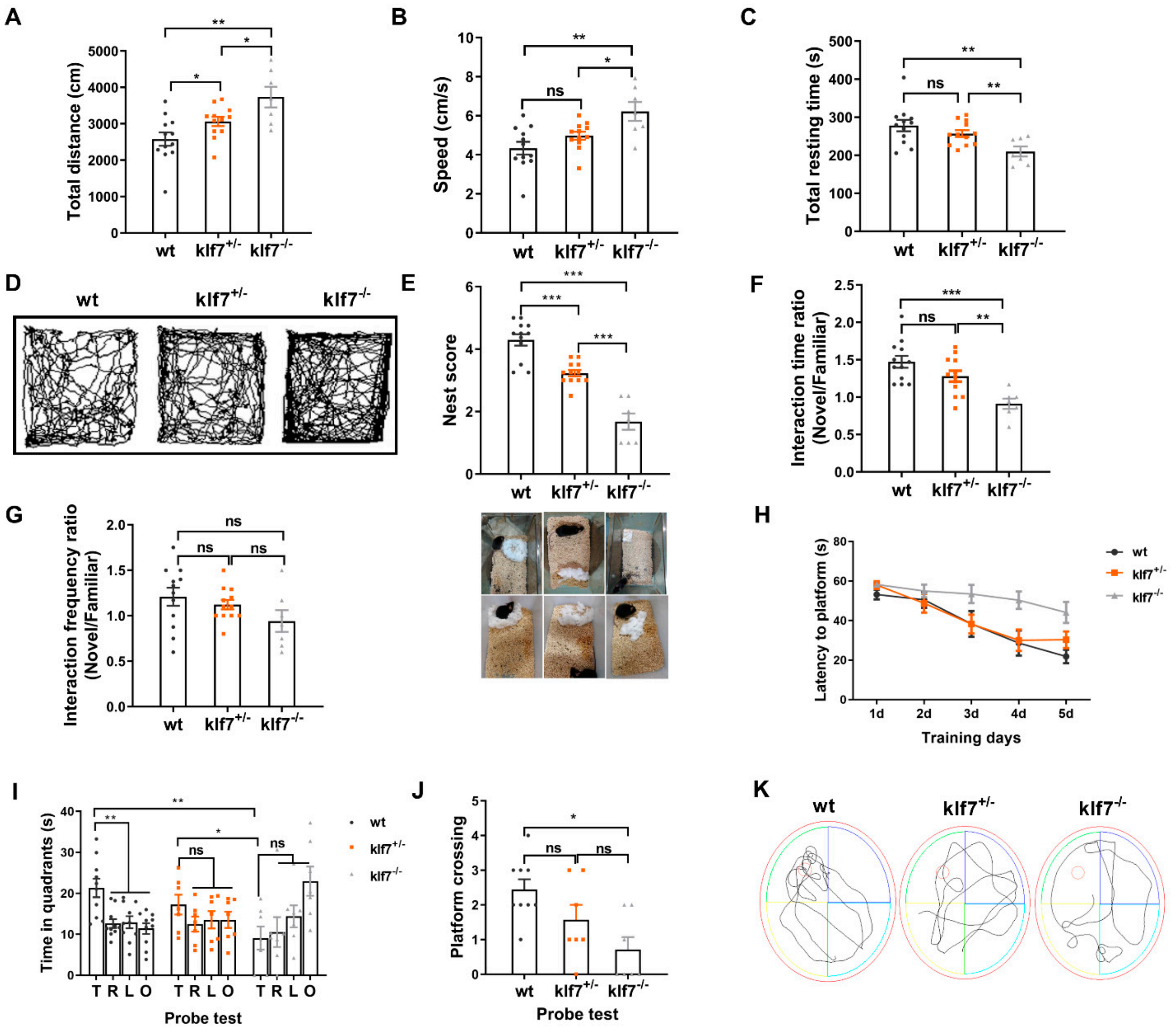

Supplemental_Fig_S4: klf7 deficient mice show other ASD-associated behaviors (related to Figure 2).

(A-D) Behavior of WT mice, klf7+/- mice and klf7-/- mice in the open field test. klf7+/- and klf7-/- mice traveled longer distances (A) and faster (B) than WT mice across the entire session. (C) WT, klf7+/-, and klf7-/- mice spent nearly the same amount of time resting. (D) Representative tracks showing the activity of WT mice, klf7+/- mice and klf7-/- mice in the open field test. (E) Nest-building test. The nesting scores of klf7+/- mice and klf7-/- mice were significantly lower than that of WT mice, as shown in the representative pictures. (F-G) Novel object recognition test. According to the ratio of the time spent interacting with the novel object to the time spent interacting with the familiar object, klf7-/- mice were able to distinguish the novel and familiar objects; however, there was no difference in the interaction time ratio between the WT and klf7+/- mice. The ratio of the number of interactions with the novel object to the number of interactions with the familiar object was not significantly different

between WT, klf7+/- and klf7-/- mice. (H-K) The Morris water maze test. (H) The spatial learning ability of WT, klf7+/-, and klf7-/- mice was assessed as the latency to find the target. klf7-/- mice showed the worst learning on the 5 training days. (I) Whereas WT mice obviously spent more time in the target region than the other three regions in the probe test, klf7+/- and klf7-/- mice spent less time in the target region than WT mice. (J) Whereas WT mice obviously crossed the platform, klf7+/- and klf7-/- mice had difficulty crossing the platform than WT mice. (K) Representative tracks showing the activity of WT, klf7+/-, and klf7-/- mice in the Morris water maze. The data are presented as the mean ± SEM. WT mice and klf7+/- mice (n = 12), klf7-/- mice (n = 7). P*<0.05, P**<0.01, P***<0.001 by one-way ANOVA and two-way ANOVA. T, target region; R, region to the right of the target right; L, region to the left of the target region; O, region opposite the target region. klf7, krüppel-like transcription factor 7; ASD, autism spectrum disorder; WT, wild type.

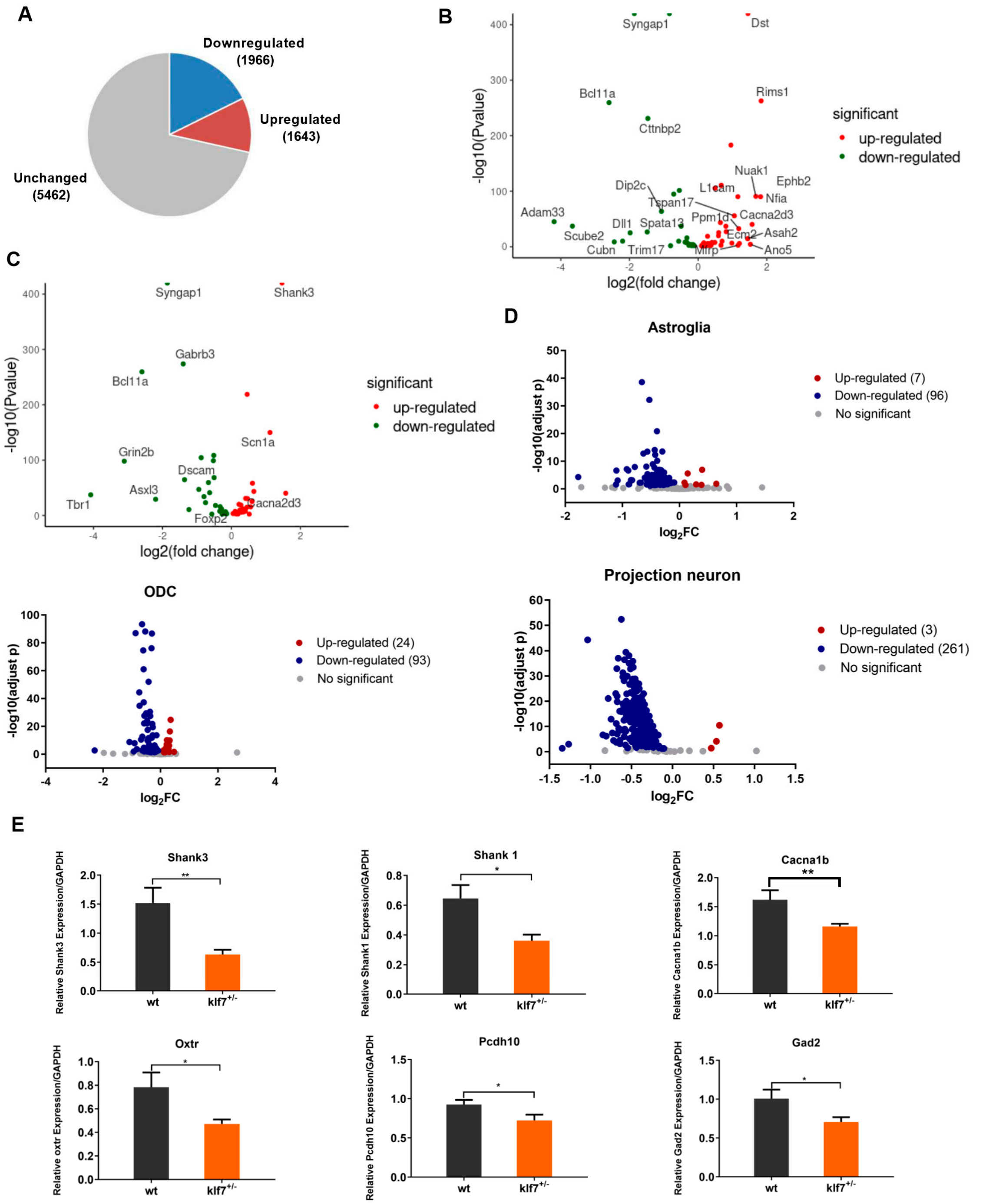

Supplemental_Fig_S5: Validation of selected DEGs by qRT-PCR (related to Figure 3).

(A) RNA-seq analysis confirmed DEGs in klf7+/- mice. (B) ASD risk genes identified by Willsey et al. were dysregulated in klf7+/- mice. Names of genes with a log2FC > 1 or log2FC< -1. (C) ASD risk genes identified by exon sequencing were dysregulated in klf7+/- mice. Names of genes with a log2FC > 1 or log2FC < -1. (D) The plot shows ASD-related DEGs in klf7+/- mice with expression patterns that were similar to those in the brains of ASD patients, and the number of genes with the same expression pattern in each cell type are presented. (E) Validation of the expression of high confidence ASD risk

genes by qRT-PCR. The data are presented as the mean ± SEM. n=9. P*<0.05, P**<0.01 by unpaired t test. DEGs, differentially expressed genes; qRT-PCR, quantitative real-time polymerase chain reaction; ASD, autism spectrum disorder; log2FC, log2(Fold Change)

A

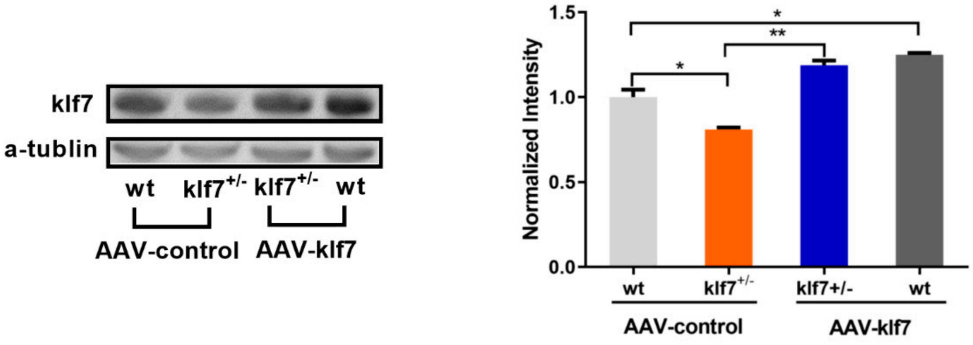

B

N	Symbol	log₂FC AAV-NC	log₂FC AAV-KLF7	State	N	Symbol	log₂FC AAV-NC	log₂FC AAV-KLF7	State
1	Chkb	3.2186626	-0.375591	Down	1	Cdh13	-1.002861	0.7403599	Up
2	Aldh1a3	2.4811969	-0.868721		2	Adcy5	-1.123202	0.4610842	
3	En2	2.254721	-3.01462		3	Drd1	-1.146532	-0.182905	
4	Tdo2	2.0240151	-0.718676		4	Oxtr	-1.152518	0.615083	
5	Ebf3	1.8977639	-0.793047		5	Col28a1	-1.278318	1.5563489	
6	Msx2	1.4636978	-2.675785		6	Dpyd	-1.42447	1.0837426	
7	Grid2	1.3575542	-1.618241		7	Pik3r2	-1.457624	1.0737576	
8	Vwa7	1.3064339	-1.920796		8	Dlx6	-1.462199	0.4967725	
9	Chd7	1.1401142	-1.502446		9	Ace	-1.544949	0.3226309	
10	Agtr2	1.1305376	-0.75177		10	Ntng1	-1.692077	1.1703859	
11	Pax6	1.1052546	-1.653038		11	Adora2a	-1.744792	-0.952222	
12	Nrxn2	1.0787003	-1.118973		12	Slc6a3	-5.636818	3.7223472	
13	Rims1	0.372466	-3.385141		13	Mfrp	-5.925447	3.0468183	

Supplemental_Fig_S6: AAV-PHP.eB-klf7 efficiently transduced cells in (related to Figure 4).

(A) Protein levels of klf7 were increased in both WT mice and klf7+/- mice administrated with AAV-PHP.eB-klf7. (B) The table shows that some of the autism-related genes showed marked changes in expression after AAV-klf7 injection. The data are presented as the mean ± SEM, n=6 for each group. P*<0.05, P**<0.01 by unpaired t test for (A). AAV, adeno-associated virus; klf7, krüppel-like transcription factor 7.
